# Integrating systemic inflammation biomarker and clinical predictors for surgical site infection risk assessment in closed pilon fractures: A risk prediction model

**DOI:** 10.1371/journal.pone.0346298

**Published:** 2026-04-06

**Authors:** Lin Jin, Yanci Zhang, Jiale Li, Lianxin Song, Yang Luo, Tianhua Dong, Xuebin Zhang

**Affiliations:** 1 Department of Orthopaedic Surgery, Hebei Medical University Third Hospital, Shijiazhuang, Hebei, People’s Republic of China; 2 Trauma Center, Hebei Medical University First Hospital, Shijiazhuang, Hebei, People’s Republic of China; Faculty of Medicine Vajira Hospital, Navamindradhiraj University, THAILAND

## Abstract

**Background:**

Systemic inflammation biomarkers have emerged as promising tools for predicting infection-related complications in orthopedic surgery. However, its predictive value for surgical site infection (SSI) after closed pilon fractures remains underexplored. This study aimed to develop and validate a nomogram that integrates systemic inflammation biomarkers and conventional clinical predictors to estimate the risk of SSI after closed pilon fracture surgery.

**Methods:**

We retrospectively analyzed data from patients aged ≥18 years with closed pilon fractures treated surgically at a tertiary orthopedic center between January 2020 and December 2023. Systemic inflammation response index (SIRI) and other candidate biomarkers were calculated from peripheral blood samples collected upon admission. The diagnosis of SSI was based on CDC criteria, determined through inpatient records and routine 12-month postoperative follow-up. Restricted cubic spline (RCS) curves were used to assess dose-response relationships between biomarkers and SSI. Multivariable logistic regression was performed to identify independent predictors and construct a nomogram. Model performance was evaluated using discrimination, calibration, and decision curve analysis (DCA). Temporal validation was performed in an independent cohort from the same center (January 2024 to December 2024), and external validation was conducted in an independent cohort from another institution (August 2024 to September 2025) using identical eligibility criteria.

**Results:**

Among 1314 patients included in model development, 57 cases (4.34%) of SSI were recorded. RCS analysis revealed a near-linear association between SIRI and SSI risk, with a threshold of 2.01 used for stratification. Multivariable analysis identified SIRI ≥ 2.01, BMI, surgical delay ≥ 6 days, Tscherne classification grade 3, prolonged surgical duration, and elevated fasting blood glucose (FBG) as independent predictors. The nomogram demonstrated good discrimination in the development cohort (AUC = 0.765) and maintained performance in temporal validation (AUC = 0.788) and external validation (AUC = 0.779).

**Conclusions:**

This study identified SIRI as a novel and independent systemic inflammation biomarker associated with SSI after closed pilon fracture. We further developed a nomogram combining SIRI and conventional clinical factors and validated it in both temporal and external cohorts, which may support individualized perioperative decision-making after further prospective multicenter validation.

## Introduction

Pilon fractures are relatively rare but severe injuries involving the distal articular surface of the tibia, accounting for approximately 1% of all lower extremity fractures and 3%–10% of tibial fractures [1,2]. These injuries are typically caused by high-energy trauma and they are often accompanied by substantial soft tissue damage, which complicates management and increases the risk of adverse outcomes [3]. Surgical treatment remains the primary approach for closed pilon fractures, but despite advancements in surgical technique and perioperative care, complications such as delayed healing, malunion, and surgical site infections (SSI) remain common [4]. SSI is one of the most devastating complications following pilon fracture surgery, with reported incidence ranging from 8.9% to 26.7% in recent studies [5–7]. Infections not only prolong hospital stay and increase healthcare costs but may also lead to reoperations, implant failure, chronic osteomyelitis, and even amputation in severe cases [8]. Therefore, early identification of patients at high risk for SSI is essential for targeted prevention and intervention.

Despite continuous improvements in surgical techniques and perioperative infection control, predicting SSI following pilon fracture surgery remains a significant clinical challenge. Previous studies have identified several conventional risk factors, such as surgical delay, operative time and the severity of soft tissue injury (e.g., Tscherne classification), as potential predictors of postoperative infection risk [4,9–11]. However, most of these models rely predominantly on static clinical indicators and often fail to capture the patient’s dynamic systemic inflammatory and nutritional status. In addition, their generalizability is limited by small sample sizes, lack of external validation and suboptimal clinical interpretability. Recently, systemic inflammation biomarkers (e.g., neutrophil-to-lymphocyte ratio [NLR], platelet-to-lymphocyte ratio [PLR], platelet-to-albumin ratio [PAR], systemic immune-inflammation index [SII], systemic inflammation response index [SIRI] and high-sensitivity C-reactive protein-to-lymphocyte ratio [HCLR]) have been increasingly recognized for their prognostic potential in predicting infectious and other postoperative complications in the context of orthopedic surgery [12–15]. The rationale behind these composite indices is that specific combinations of circulating cells and proteins can more accurately mirror adverse systemic physiological states than any single parameter alone. For example, as primary innate effector cells, perioperative expansion of neutrophils and monocytes indicates excessive activation of the systemic inflammatory response, accompanied by the release of proteases, reactive oxygen species and pro-inflammatory cytokines that may damage endothelial cells and impair microcirculatory perfusion [16]. In contrast, circulating lymphopenia is a hallmark of surgery- and trauma-induced immunosuppression and has been associated with heightened susceptibility to nosocomial and surgical infections [17]. Taken together, an elevated SIRI, which simultaneously captures neutrophilia/monocytosis and relative lymphopenia, reflects a state of “inflammation present but impaired immune function”. In such a state, bacterial clearance at the surgical site is likely compromised and the risk of postoperative infection is increased [15]. These markers, derived from routine blood tests, have demonstrated promising predictive performance in patients undergoing surgery and may reflect an individual’s immune resilience and infection susceptibility more objectively. Therefore, integrating systemic inflammation biomarkers into existing predictive frameworks may significantly enhance risk stratification for SSI after closed pilon fracture, offering more actionable insights for individualized perioperative management.

Given this context, the present study aims to systematically evaluate the prognostic performance of systemic inflammation biomarkers for SSI after closed pilon fracture surgery. By integrating these markers with traditional clinical variables, we sought to construct and validate a clinically practical nomogram for individualized SSI risk stratification.

## Materials and methods

### Study design and populations

This was a retrospective study with temporal validation in the same center and external validation in an independent center. Patients were included if they: (1) were aged ≥18 years; (2) had a confirmed diagnosis of pilon fracture based on the Rüedi and Allgöwer classification; and (3) underwent surgical treatment at the participating institutions. Exclusion criteria included: (1) open or pathological fractures; (2) multiple fracture; (3) preoperative systemic or local infection; (4) history of autoimmune disease, malignancy, or immunosuppressive therapy; (5) incomplete or missing data; (6) loss to follow-up. All patients received standardized perioperative antibiotic prophylaxis according to institutional protocol. Patients who underwent surgery between January 2020 and December 2023 at our institution were included in the development cohort. Temporal validation was conducted using patients treated at the same institution between January 2024 and December 2024. External validation was conducted using patients treated at another institution between August 2024 and September 2025, retrospectively screened using the same eligibility criteria.

Data for model development and temporal validation were accessed for research purposes on January 15, 2025. The institutional ethics committee approved the study (approval number: W2024-021-1; 2024-S01046) and waived the requirement for informed consent owing to its retrospective design and de-identification of patient data. Data for external validation were accessed for research purposes on December 24, 2025. The external validation cohort was approved by the ethics committee of the participating external institution (approval number: 2024-S01046), with informed consent similarly waived. The study was conducted in accordance with the Declaration of Helsinki [18] and the Strengthening the Reporting of Cohort Studies in Surgery (STROCSS) guidelines [19]. During or after the data collection period, the authors did not have access to any information that could identify individual participants.

### General information

All general Information for this study were obtained by well-trained researchers who were independent of patient care. Clinical information was extracted from electronic medical records (EMR system, Kaihua Network Technology Co., Ltd., Beijing), imaging data were retrieved from the picture archiving and communication system (PACS, iMedical, DHC Software Co., Ltd., Beijing), and laboratory test results as well as microbiological culture records were obtained from the laboratory information system (LIS, RMLIS, Rui Mei Computer Technology Co., Ltd., Shanghai).

Preoperative baseline data included demographic and injury-related variables: age, sex, body mass index (BMI), residence, alcohol use, smoking status, Charlson Comorbidity Index (CCI), comorbidities (hypertension, diabetes, cardiovascular disease, heart disease, chronic respiratory disease, liver disease, kidney disease, and malignancy), mechanism of injury, surgical delay (the time from injury to the index operation, calculated as the number of days between the documented date and time of injury and the date of definitive fixation), Rüedi and Allgöwer classification, Tscherne soft tissue classification, and American Society of Anesthesiologists (ASA) score.

Perioperative variables included anesthesia method, surgical duration, intraoperative blood loss, surgical fixation methods, surgical approach, bone grafting, type of prophylactic antibiotics administered, and postoperative antibiotic usage.

### Acquisition and derivation of the systemic inflammation biomarkers

Fasting blood samples used to derive systemic inflammation biomarkers were collected from peripheral veins on the first morning after admission and before any surgical procedure [20]. Because only patients with closed pilon fractures undergoing elective definitive fixation were included, no emergency surgeries were performed before this standardized preoperative sampling. All laboratory assays were conducted following the manufacturers’ protocols. Biochemical parameters were measured using the Beckman Coulter AU5800 chemistry analyzer, while hematological parameters were obtained using the UniCel DXI 800 system (Beckman Coulter).

Reported predictive biomarkers, including white blood cells (WBC), neutrophils (NEU), lymphocytes (LYM), serum albumin (ALB), erythrocyte sedimentation rate (ESR), high-sensitivity C-reactive protein (HCRP) and fasting blood glucose (FBG), were included for assessment.

Potential systemic inflammation biomarkers were calculated using the following formulas: SII = Platelet count (×10⁹/L) × Neutrophil count (×10⁹/L)/ Lymphocyte count (×10⁹/L); SIRI = Neutrophil count (×10⁹/L) × Monocyte count (×10⁹/L)/ Lymphocyte count (×10⁹/L); NLR = Neutrophil count (×10⁹/L)/ Lymphocyte count (×10⁹/L); PLR = Platelet count (×10⁹/L)/ Lymphocyte count (×10⁹/L); HCLR = HCRP (mg/L)/ Lymphocyte count (×10⁹/L); PAR = Platelet count (×10⁹/L)/ Albumin (g/L).

### Surgical procedures and soft-tissue management

All operations were performed by experienced orthopedic trauma surgeons using standard open/closed reduction and internal fixation techniques. Surgical approaches were selected according to fracture morphology and soft-tissue conditions and were documented as either single-incision or multiple-incision procedures; these variables were compared between the SSI and non-SSI groups. All injuries in this cohort were closed pilon fractures. Definitive fixation was undertaken only after soft-tissue swelling had subsided and skin wrinkling had returned. Although a proportion of patients presented with severe soft-tissue contusion (Tscherne grade 3), all surgical incisions could be closed primarily or with simple tension-reducing sutures, and no patient required rotational or free flap coverage. Consequently, there were no differences in flap procedures that might confound the association between preoperative systemic inflammatory indices and SSI.

### Perioperative antibiotic prophylaxis

Perioperative antibiotic prophylaxis followed a standardized institutional protocol based on WHO guidelines [21]: intravenous antibiotics were administered within 30 minutes before skin incision and routinely discontinued within 24 hours after surgery. Cefazolin (first-generation cephalosporin) was the primary agent, with cefuroxime, ceftriaxone or clindamycin used as alternatives when indicated (e.g., recent alcohol use or β-lactam allergy). Antibiotic type and postoperative duration (days) were recorded and compared between SSI and non-SSI groups.

### Diagnosis of SSI

The diagnostic criteria for SSI in this study were based on the definitions from the Centers for Disease Control and Prevention (CDC). Superficial SSI was defined as signs of erythema, localized swelling, tenderness, or fever involving the skin or subcutaneous tissue within 30 days postoperatively, typically managed with wound care and oral antibiotics. Deep SSI involved deeper soft tissue structures (e.g., fascia or muscle) within 90 days after surgery, characterized by persistent wound discharge, dehiscence, abscess formation, or necrosis, and often requiring surgical debridement, systemic antibiotics, or implant management [22].

All inpatient medical records, laboratory pathogen culture results, and imaging studies were comprehensively reviewed. To ensure complete identification of SSI cases, patients were routinely followed up by telephone for more than 12 months postoperatively. This long-term follow-up allowed detection of infections diagnosed or treated at other institutions beyond the primary observation window. For patients who reported an SSI during follow-up but lacked corresponding documentation within our hospital system, written confirmation from the treating external institution was requested. For each reported episode, the timing of symptom onset and treatment was checked to determine whether it fell within the 30- or 90-day CDC windows; only those events were classified as SSI and included in the primary outcome. Infections that first occurred beyond 90 days were not counted as SSI in the main analysis. The determination of SSI was independently performed by two orthopedic specialists, with disagreements resolved through discussion with a senior chief orthopedic surgeon.

### Statistical analysis

Patients with missing key baseline, laboratory, or outcome data, or those lost to follow-up, were excluded from both the development and validation cohorts; therefore, all analyses were conducted on complete cases, and no imputation for missing values was performed. Continuous variables were presented as mean ± standard deviation or median [Q1–Q3], according to their distribution, and compared using Student’s t test or the Mann–Whitney U test, as appropriate. Categorical variables were expressed as numbers (%) and analysed with the chi-square test or Fisher’s exact test. In line with international guidelines that recommend 48 h as a cut-off for early surgery, we examined both 48 h and 5 days as thresholds for surgical delay [23].

To explore potential linear or non-linear associations between systemic inflammation biomarkers and SSI risk, restricted cubic spline (RCS) analyses with four knots were applied to biomarkers that were statistically significant (*P* < 0.05) in univariate logistic regression, using R software (version 4.3.2) and the rms, ggrcs and ggplot2 packages. RCS models with four knots were fitted for SIRI and HCLR, with knots located at the approximately 5th, 35th, 65th and 95th percentiles of the distribution. RCS curves were used to visualize dose-response patterns and identify potential thresholds or inflection points. The inflection point was pragmatically defined as the value at which the predicted relative risk was closest to 1. Computationally, this was implemented by calculating the absolute difference between the RCS-based predicted relative risk and 1 across all evaluated values and selecting the value with the minimum difference. Biomarkers with significant trends and plausible dose-response relationships in the RCS analysis, along with other potential predictors with *P* < 0.05 in univariate analysis, were included in multivariable logistic regression models. Backward stepwise selection was used to identify independent predictors of SSI. Prior to modeling, multicollinearity was assessed using the variance inflation factor (VIF), and variables with VIF ≥ 3 were excluded to ensure model stability [24].

Finally, a prognostic nomogram was developed to predict the risk of SSI, incorporating both the selected systemic inflammation biomarkers and conventional clinical predictors. The model’s discriminative ability was assessed using the receiver operating characteristic (ROC) curve, with the area under the curve (AUC) and concordance index (C-index) reported. Higher AUC and C-index values (closer to 1.0) indicate better discriminatory performance. Calibration performance was evaluated by plotting calibration curves to compare the predicted probabilities with the observed outcomes. The Hosmer-Lemeshow goodness-of-fit test was used to statistically assess calibration, and the Brier score was calculated to quantify overall prediction accuracy, with lower values indicating better calibration. The clinical utility of the nomogram was assessed using decision curve analysis (DCA), which estimates the net clinical benefit across a range of threshold probabilities. Internal validation was conducted using the bootstrap resampling method (1,000 iterations) to obtain optimism-corrected estimates of the C-index and Brier score. Model validation included bootstrap internal validation, temporal validation in an independent cohort from the same center, and external validation in an independent cohort from another institution.

As this was a retrospective study including all consecutive eligible patients over the study period, no formal a priori sample-size or power calculation was performed. The development cohort comprised 57 SSI events and 1,257 non-events, yielding approximately nine events per predictor in the final multivariable logistic regression model, which we considered acceptable according to contemporary recommendations for prediction modelling [25]. ROC analyses used the development cohort and two independent validation cohorts, including a temporal validation cohort from the same center (n = 562) and an external validation cohort from another center (n = 359), to assess model discrimination.

For SIRI, which showed an approximately linear increase in SSI risk above about 2.01 on the RCS curve, the variable was additionally dichotomized at 2.01 (low < 2.01 vs high ≥ 2.01) to facilitate clinical interpretation, and the robustness of this pre-specified cut-off was examined using bootstrap resampling with 1,000 iterations (Supplementary S1 Table). In exploratory analyses, ROC curves were also constructed for individual systemic inflammation biomarkers (SII, SIRI, NLR, PLR, HCLR and PAR), and pairwise DeLong tests were used to formally compare their AUCs, with SIRI specified as the reference curve; results are summarized in Supplementary S1 Table.

We conducted a sensitivity analysis for SIRI, in which the continuous variable was dichotomized at 2.01 (low < 2.01 vs high ≥ 2.01), corresponding to the value at which the RCS-based predicted relative risk of SSI was closest to 1, in order to facilitate clinical interpretation. The robustness of this RCS-derived cut-off was evaluated using bootstrap resampling with 1,000 iterations. In a further sensitivity analysis, a baseline logistic regression model including only conventional clinical predictors (BMI, surgical delay, Tscherne classification, surgical duration and FBG) was fitted and its discriminative performance (AUC) was compared with that of the full model that also incorporated dichotomized SIRI using ROC analysis. Furthermore, we conducted exploratory analyses in which ROC curves were constructed for individual systemic inflammation biomarkers (SII, SIRI, NLR, PLR, HCLR and PAR), and pairwise DeLong tests were used to formally compare their AUCs, with SIRI specified as the reference curve.

All statistical analyses were conducted using R software (version 4.3.2; R Foundation for Statistical Computing), and a two-sided *P* value < 0.05 was considered statistically significant.

## Results

### Clinical characteristics

**Fig 1:**
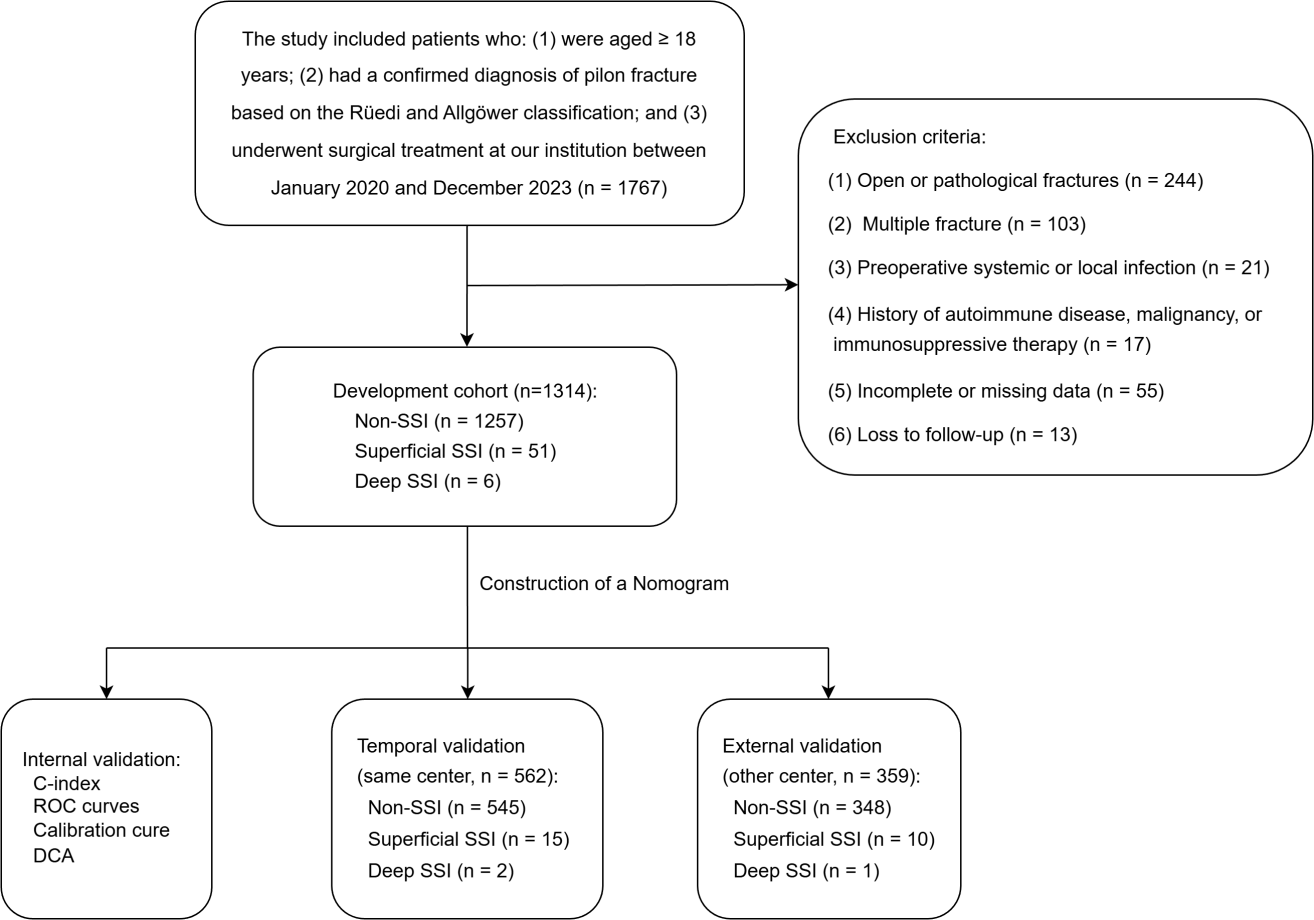
Flow diagram of patient selection and cohort allocation. SSI, surgical site infection; ROC, receiver-operating characteristic; DCA, decision curve analysis.

Based on the predefined inclusion and exclusion criteria, a total of 1,314 patients were enrolled for model development (Fig 1). The cohort had a median age of 40 years (interquartile range [IQR]: 27–58), and 72.0% (n = 947) were male. The median BMI was 25.5 kg/m² (IQR: 23.2–27.6). Among the included cases, 57 patients (4.34%) developed SSIs, of which 51 (3.88%) were classified as superficial infections and 6 (0.46%) as deep infections according to the CDC definition (Table 1). For patients with multiple SSI events, only the most severe episode was considered for analysis. A temporal validation cohort comprising 562 patients was identified from the same center, with 17 cases of SSI (3.02%), including 15 (2.67%) superficial and 2 (0.36%) deep infections (Fig 1). In addition, an external validation cohort from another institution comprised 359 patients, including 11 SSI cases (3.06%), with 10 superficial and 1 deep infection (Fig 1).

**Table 1:** Baseline characteristics of the study population^•^.

Characteristic	Overall N = 1,314	Non-SSI group N = 1,257	SSI group N = 57	*P*
**Age**	40.00 [27.00, 58.00]	40.00 [27.00, 58.00]	44.00 [33.00, 65.00]	0.046*
**Sex**				0.287
Man	947 (72.1%)	902 (71.8%)	45 (78.9%)	
Woman	367 (27.9%)	355 (28.2%)	12 (21.1%)	
**BMI (kg/m**^**2**^)	25.45 [23.22, 27.56]	25.38 [23.19, 27.54]	26.77 [24.48, 28.19]	0.013*
**Residence**				1.000
Urban	931 (70.9%)	891 (70.9%)	40 (70.2%)	
Rural	383 (29.1%)	366 (29.1%)	17 (29.8%)	
**Alcohol use**	883 (67.2%)	838 (66.7%)	45 (78.9%)	0.052
**Currently smoking**	464 (35.3%)	436 (34.7%)	28 (49.1%)	0.031*
**CCI**	1.00 [0.00, 2.00]	1.00 [0.00, 2.00]	1.00 [0.00, 2.00]	0.718
0	364 (27.7%)	348 (27.7%)	16 (28.1%)	0.889
1-2	659 (50.2%)	629 (50.0%)	30 (52.6%)	
≥ 3	291 (22.1%)	280 (22.3%)	11 (19.3%)	
**Preoperative comorbidities**				
Hypertension	349 (26.6%)	334 (26.6%)	15 (26.3%)	1.000
Diabetes	115 (8.8%)	106 (8.4%)	9 (15.8%)	0.054
Cerebrovascular disease	69 (5.3%)	65 (5.2%)	4 (7.0%)	0.522
Heart disease	95 (7.2%)	88 (7.0%)	7 (12.3%)	0.178
Chronic respiratory disease	6 (0.5%)	6 (0.5%)	0 (0.0%)	1.000
Liver disease	46 (3.5%)	45 (3.6%)	1 (1.8%)	0.703
Kidney disease	51 (3.9%)	47 (3.7%)	4 (7.0%)	0.264
Malignancy	18 (1.4%)	17 (1.4%)	1 (1.8%)	1.000
**Mechanism of injury**				0.238
High falling	441 (33.5%)	424 (33.7%)	17 (29.8%)	
Traffic injury	453 (34.5%)	437 (34.8%)	16 (28.1%)	
Fall down	420 (32.0%)	396 (31.5%)	24 (42.1%)	
**Surgical delay (days)**				0.003*
< 2	751 (57.2%)	730 (58.1%)	21 (36.8%)	
2-5	296 (22.5%)	280 (22.3%)	16 (28.1%)	
≥ 6	267 (20.3%)	247 (19.6%)	20 (35.1%)	
**Rüedi and Allgöwer classification**				0.075
I	219 (16.7%)	211 (16.8%)	8 (14.0%)	
II	526 (40.0%)	510 (40.6%)	16 (28.1%)	
III	569 (43.3%)	536 (42.6%)	33 (57.9%)	
**Tscherne classification**				0.002*
Grade 0	216 (16.4%)	211 (16.8%)	5 (8.8%)	
Grade 1	590 (44.9%)	572 (45.5%)	18 (31.6%)	
Grade 2	331 (25.2%)	313 (24.9%)	18 (31.6%)	
Grade 3	177 (13.5%)	161 (12.8%)	16 (28.0%)	
**ASA score**				0.539
I	136 (10.3%)	130 (10.3%)	6 (10.5%)	
II	929 (70.7%)	892 (71.0%)	37 (64.9%)	
III	247 (18.8%)	233 (18.5%)	14 (24.6%)	
IV	2 (0.2%)	2 (0.2%)	0 (0.0%)	
**Anesthesia method**				0.384
General	921 (70.1%)	878 (69.8%)	43 (75.4%)	
Regional	393 (29.9%)	379 (30.2%)	14 (24.6%)	
**Surgical duration (minutes)**	117.00 [75.00, 151.00]	116.00 [73.00, 150.00]	138.00 [86.00, 155.00]	0.025*
**Intraoperative blood loss (mL)**	132.00 [83.00, 192.00]	131.00 [82.00, 191.00]	154.00 [94.00, 206.00]	0.071
**Surgical fixation methods**				0.820
ORIF with plate	941 (71.6%)	900 (71.6%)	41 (71.9%)	
ORIF with screws	135 (10.3%)	128 (10.2%)	7 (12.3%)	
CRIF with percutaneous screws	238 (18.1%)	229 (18.2%)	9 (15.8%)	
**Surgical approach**				0.218
Single incision	954 (72.6%)	917 (73.0%)	37 (64.9%)	
Multiple incisions	360 (27.4%)	340 (27.0%)	20 (35.1%)	
**Bone graft**				0.252
No	1,192 (90.7%)	1,143 (90.9%)	49 (86.0%)	
Yes	122 (9.3%)	114 (9.1%)	8 (14.0%)	
**Antibiotics type** ^°^				0.747
1st cephalosporin	1,115 (84.9%)	1,064 (84.7%)	51 (89.5%)	
2nd cephalosporin	50 (3.8%)	49 (3.9%)	1 (1.8%)	
3rd cephalosporin	80 (6.0%)	77 (6.1%)	3 (5.2%)	
Other antibiotic	69 (5.3%)	67 (5.3%)	2 (3.5%)	
**Postoperative antibiotic use (days)**	1.00 [1.00, 2.00]	1.00 [1.00, 2.00]	1.00 [1.00, 2.00]	0.983
WBC (*10 ^**9**^ **/L)**	8.31 [6.65, 10.47]	8.30 [6.65, 10.45]	8.45 [6.63, 10.73]	0.474
NEU (*10 ^**9**^ **/L)**	4.85 [3.38, 6.90]	4.85 [3.38, 6.87]	4.81 [3.55, 7.81]	0.170
LYM (*10 ^**9**^ **/L)**	2.38 [1.60, 3.17]	2.40 [1.60, 3.17]	2.21 [1.45, 3.12]	0.314
**ALB (g/L)**	35.88 [33.00, 39.19]	35.90 [33.00, 39.20]	35.10 [31.20, 38.80]	0.201
**ESR (mm/h)**	20.81 [10.66, 30.23]	20.57 [10.57, 30.16]	22.24 [13.24, 32.15]	0.221
**HCRP (mg/L)**	5.43 [3.01, 7.97]	5.42 [2.98, 7.95]	6.61 [3.85, 8.37]	0.071
**FBG (mmol/L)**	5.81 [4.82, 6.63]	5.78 [4.80, 6.61]	6.23 [5.27, 7.15]	0.008*
**Systemic Inflammation Biomarkers**				
SII	432.50 [261.83, 647.30]	429.70 [260.33, 646.14]	484.87 [295.06, 696.83]	0.338
SIRI	2.01 [1.22, 3.14]	2.00 [1.22, 3.12]	2.59 [1.48, 3.41]	0.028*
NLR	2.20 [1.38, 3.36]	2.16 [1.38, 3.33]	2.62 [1.57, 3.70]	0.104
PLR	83.22 [61.90, 122.13]	83.12 [61.79, 122.17]	88.32 [67.43, 117.80]	0.837
HCLR	2.35 [1.29, 3.85]	2.33 [1.27, 3.82]	2.94 [1.74, 4.65]	0.029*
PAR	5.38 [4.52, 6.61]	5.38 [4.53, 6.59]	5.47 [4.15, 6.89]	0.706

Note: *Statistical significance.

• Values are median [Q1–Q3] for continuous variables and n (%) for categorical variables.

° Antibiotics were administered within 30 min before incision and discontinued within 24 h postoperatively, per WHO guidelines. Cefazolin (1st) was the primary choice, with cefuroxime (2nd), ceftriaxone (3rd), or clindamycin (other antibiotic, for recent alcohol use or beta-lactam allergy) used as alternatives. Duration was extended for specific clinical needs based on judgment.

Abbreviations: SSI, surgical site infection; BMI, body mass index; ASA, American society of anesthesiologists; CCI, Charlson comorbidity index; ORIF, open reduction and internal fixation; CRIF, closed reduction and internal fixation; WBC, white blood cell; NEU, neutrophil; LYM, lymphocyte; ALB albumin; ESR, erythrocyte sedimentation rate; HCRP, high-sensitivity C-reactive protein; FBG fasting blood glucose; SII, systemic immune inflammation index; SIRI, System inflammation response index; NLR, neutrophil-to-lymphocyte ratio; PLR, platelet-to-lymphocyte ratio; HCLR, high-sensitivity C-reactive protein-to-lymphocyte ratio; PAR, platelet-to-albumin ratio.

### Exploratory analysis for biomarker selection prior to modeling

As shown in Table 1, univariate logistic regression identified two systemic inflammation biomarkers—SIRI (*P* = 0.028) and HCLR (*P* = 0.029)—as significantly associated with the occurrence of SSI. These variables were further analyzed using RCS modeling with four knots to explore their potential linear or non-linear associations with SSI risk.

**Fig 2:**
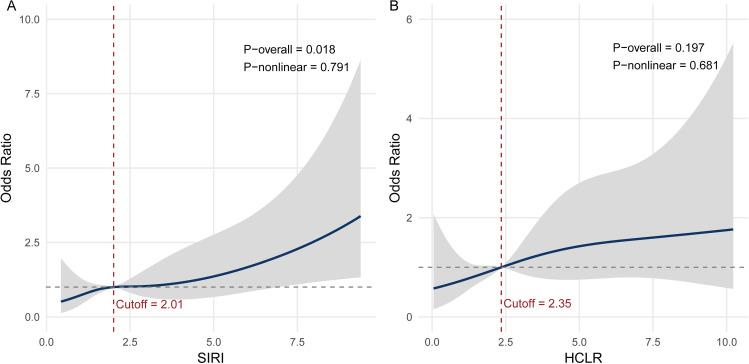
Restricted cubic spline (RCS) curves for the association between systemic inflammation biomarkers and the risk of surgical site infection (SSI). **(a)** The SIRI curve demonstrated a significant linear association with SSI (P-overall = 0.018; P-nonlinear = 0.791), with a threshold cutoff value of 2.01 indicated by the vertical red dashed line. **(b)** The HCLR curve did not show a statistically significant association with SSI (P-overall = 0.197; P-nonlinear = 0.681), suggesting weaker predictive value.

The RCS curve for SIRI revealed a statistically significant overall association with SSI (*P*-overall < 0.05), and the *P* for non-linearity (*P*-nonlinear) was > 0.05, suggesting that the relationship between SIRI and SSI is approximately linear across its range (Fig 2). In contrast, HCLR showed neither significant overall nor non-linear associations with SSI (both *P*-overall and *P*-nonlinear > 0.05).

Based on the statistical significance in univariate analysis and the observed dose-response trend from the RCS model, SIRI was selected as the final systemic inflammation biomarker to be included in the subsequent multivariable logistic regression model. To facilitate clinical interpretability and improve model robustness, SIRI was dichotomized using an RCS-derived threshold of 2.01, with patients stratified into low-SIRI (< 2.01) and high-SIRI (≥ 2.01) groups for all subsequent analyses.

### Univariate and multivariable analysis

In the univariate analysis, several clinical variables—including age, BMI, currently smoking, surgical delay, Tscherne classification, surgical duration, and FBG—were significantly associated with the risk of SSI (all *P* < 0.05). These variables, along with the previously selected systemic inflammation biomarker SIRI, were entered into a multivariable logistic regression model. Variable selection was performed using backward stepwise elimination based on the minimum Akaike Information Criterion (AIC). No multicollinearity was detected among the variables. The final model identified the following as independent risk factors for SSI: elevated BMI, surgical delay ≥ 6 days, Tscherne grade 3, longer surgical duration, higher FBG, and SIRI ≥ 2.01 (Table 2).

**Table 2:** Multivariable analyses of the independent risk factors associated with postoperative SSI.

Variables	P value	Odds Ratio	95% CI
**BMI (kg/m^2^)**	0.008*	1.12	1.03-1.23
**Surgical delay (days)**			
<2	1.000		
2-5	0.156	1.65	0.83-3.30
≥6	0.005*	2.57	1.34-4.96
**Tscherne classification**			
Grade 0	1.000		
Grade 1	0.465	1.46	0.53-4.07
Grade 2	0.060	2.69	0.96-7.55
Grade 3	0.002*	5.34	1.86-15.34
**Surgical duration (minutes)**	0.001*	1.01	1.00-1.02
**FBG (mmol/L)**	0.003*	1.37	1.12-1.69
**SIRI** <2.01 ≥2.01	0.027*	1.97	1.08-3.58

Note: *Statistical significance.

Abbreviations: SSI, surgical site infection; OR, odd ratio; CI, confidence interval; BMI, body mass index; FBG fasting blood glucose; SIRI, System inflammation response index.

### Construction and validation of a nomogram

**Fig 3:**
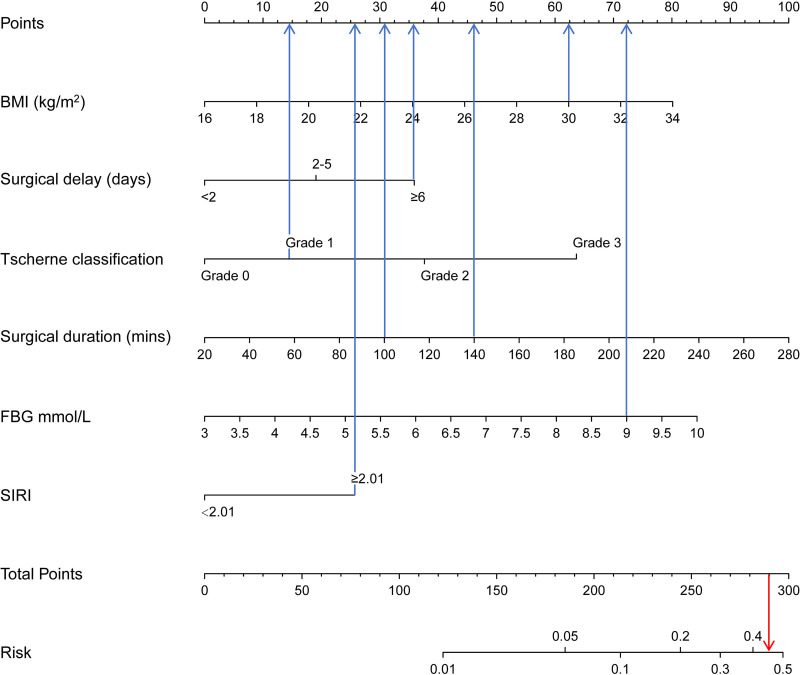
Nomogram for predicting the risk of SSI in patients with closed pilon fractures. The nomogram incorporates six predictors: BMI, surgical delay, Tscherne classification, surgical duration, fasting blood glucose (FBG), and SIRI (categorized by cutoff value of 2.01). Each variable contributes a point score, and the total score corresponds to an estimated probability of SSI.

A predictive nomogram was constructed based on the six independent risk factors identified in the multivariable logistic regression analysis. As illustrated in Fig 3, the nomogram allows users to draw vertical lines corresponding to each predictor’s value to determine its individual point contribution (blue line), sum these to obtain a total score, and then project a vertical line downward to estimate the predicted probability of SSI (red line).

**Fig 4:**
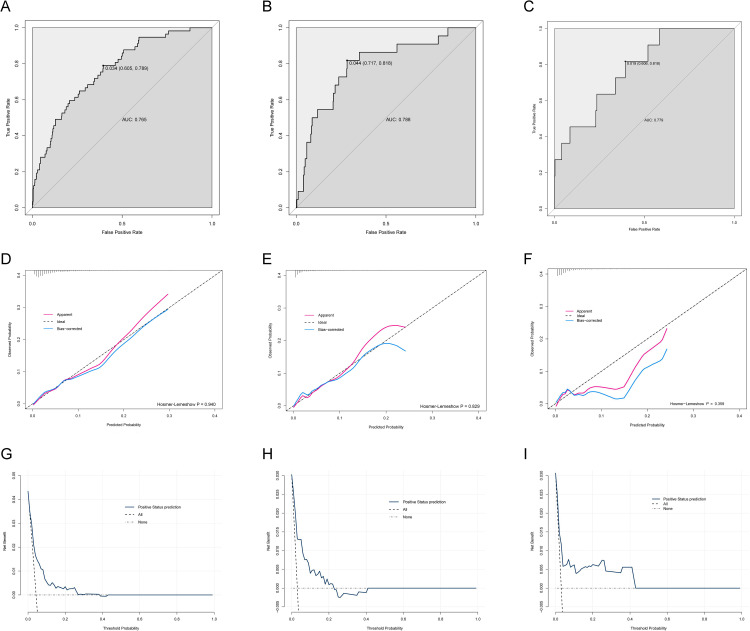
(A–C) Receiver operating characteristic (ROC) curves for model discrimination in the development cohort (A), temporal validation cohort from the same center (B), and external validation cohort from another institution (C). The nomogram achieved an AUC of 0.765 (95% CI: 0.705–0.825) with a sensitivity of 60.5% and specificity of 78.9% in the development cohort; an AUC of 0.788 (95% CI: 0.717–0.818) with a sensitivity of 71.7% and specificity of 81.8% in the temporal validation cohort; and an AUC of 0.779 (95% CI: 0.649–0.908) with a sensitivity of 60.6% and specificity of 81.8% in the external validation cohort. (D–F) Calibration curves of the nomogram in the development (D), temporal validation (E), and external validation (F) cohorts, demonstrating agreement between predicted and observed risks (Hosmer–Lemeshow P = 0.940, 0.829, and 0.359, respectively). Apparent, bias-corrected, and ideal lines are shown. (G–I) Decision curve analysis (DCA) evaluating clinical utility in the development (G), temporal validation (H), and external validation (I) cohorts. The nomogram yielded a positive net benefit across threshold probabilities of approximately 0.5%–26% in the development cohort, 0.4%–22% in the temporal validation cohort, and 0.4%–43% in the external validation cohort.

The model demonstrated good discriminative performance, with an AUC of 0.765 (95% confidence interval [CI]: 0.705–0.825), a specificity of 78.9%, and a sensitivity of 60.5% (Fig 4A). The C-index and Brier score of the model were 0.765 and 0.039, respectively. After bootstrap internal validation with 1,000 replications, the corrected C-index and Brier score were 0.729 and 0.041, indicating favorable model stability and overall performance. It remained robust in the temporal validation cohort with an AUC of 0.788 (95% CI: 0.717–0.818) (Fig 4B) and in the external validation cohort with an AUC of 0.779 (95% CI 0.649–0.908) (Fig 4C).

Calibration was assessed in the development, temporal validation, and external validation cohorts (Fig 4D–F). The Hosmer–Lemeshow test showed non-significant P values of 0.940, 0.829, and 0.359, respectively, suggesting good agreement between predicted and observed risks. Decision curve analysis suggested that the nomogram provided net clinical benefit across clinically relevant threshold probabilities in the development cohort and both validation cohorts (Fig 4G–I).

### Sensitivity and exploratory analyses

In a sensitivity analysis, the stability of the RCS-derived dichotomization of SIRI at 2.01 was evaluated by bootstrap resampling of the development cohort (1,000 iterations). This procedure yielded a median odds ratio for high versus low SIRI of 2.21 and a median AUC of 0.595, with relatively narrow 2.5th–97.5th percentile ranges, indicating good robustness of the chosen cut-off (Supplementary S1 Table). Another sensitivity analysis, a baseline logistic regression model including only conventional clinical predictors (BMI, surgical delay, Tscherne classification, surgical duration and FBG) was fitted in the development cohort and its performance was compared with that of the full model that also incorporated dichotomized SIRI. The baseline model achieved an AUC of 0.751 (95% CI, 0.6871–0.8146), whereas the full model achieved an AUC of 0.765 (95% CI, 0.705–0.825), suggesting a modest gain in discrimination when SIRI is added to clinical variables alone (S1 Fig).

In exploratory analyses, ROC curves were generated to compare the discriminative performance of individual systemic inflammation indices in the development cohort (S2 Fig). SIRI yielded the numerically highest AUC (0.586), similar to that of HCLR (0.586), and higher than those of NLR (0.564), SII (0.537), PLR (0.508) and PAR (0.485). Pairwise DeLong tests using SIRI as the reference curve showed that its AUC was significantly higher than those of SII, PLR and PAR (*P* = 0.005, 0.009 and 0.033, respectively), whereas differences between SIRI and NLR or HCLR were not statistically significant (*P* = 0.057 and 0.496, respectively). Detailed comparisons are presented in Supplementary S2 Table.

## Discussion

In this retrospective cohort of patients with closed pilon fractures, we found that a composite of one systemic inflammation biomarker (SIRI) and five readily available clinical variables independently predicted SSI. Based on these predictors, we constructed a nomogram that showed acceptable discrimination and calibration, and provided net clinical benefit in DCA. The model also performed consistently in both temporal and external validation cohorts, supporting its robustness and suggesting potential clinical utility.

This study is the first to demonstrate the independent predictive value of systemic inflammation biomarkers for SSI following closed pilon fractures. Because open fractures are a well-established major risk factor for SSI and deep infection, particularly in higher Gustilo–Anderson grades, we restricted our cohort to closed pilon fractures to obtain a more homogeneous baseline risk profile and to better isolate the prognostic value of preoperative systemic inflammatory indices [26,27]. To comprehensively assess the association between candidate biomarkers and SSI risk, we employed RCS analysis to examine dose-response relationships for SIRI and HCLR, which were identified as significant in univariate analysis. Compared with conventional approaches such as quartile stratification or thresholds derived from Youden’s index, the RCS method offers enhanced flexibility in modeling both linear and non-linear associations and enables the identification of clinically meaningful inflection points based on the observed data distribution [28]. This approach strengthens both the biological relevance and the clinical interpretability of risk stratification. Based on the RCS findings and overall model performance, SIRI was selected as the optimal biomarker, and a threshold value of 2.01 was determined to categorize patients according to their preoperative systemic inflammatory state in subsequent analyses.

Several recent multicentre studies have incorporated systemic inflammation biomarkers into prediction models for orthopaedic infections, which provides an important context for the present work. In the periprosthetic joint infection (PJI) setting, Yu et al. demonstrated that the PAR and the C-reactive protein-to-albumin ratio were correlated with treatment failure across two orthopaedic centres, although these markers were not integrated into a preoperative risk-stratification nomogram [29]. Pang et al. employed machine-learning algorithms to incorporate composite indices, including the aggregate index of systemic inflammation (AISI), SII and the C-reactive protein–albumin–lymphocyte (CALLY) index, alongside clinical variables, achieving excellent discrimination for SSI after posterior lumbar fusion in an elective spine population across two tertiary hospitals [30]. SIRI has been increasingly recognised as a robust prognostic indicator in various clinical contexts, including orthopaedic trauma and surgery [31–38]. In elderly hip fracture cohorts, elevated SIRI has been associated with higher long-term mortality and poorer functional recovery, with restricted cubic spline analyses supporting a dose–response relationship with adverse outcomes [31,32]. In addition, Vitiello et al. reported that lower SIRI values at explantation and greater perioperative reductions (delta-SIRI) were significantly associated with infection resolution in patients with chronic PJI, suggesting that SIRI reflects clinically relevant immune–inflammatory dynamics in bone-related infections, although this study was limited by its small sample size (n = 57) [39]. Collectively, these data support the biological plausibility of SIRI as a marker of perioperative immune dysregulation that may influence infection risk. Several prediction tools for SSI after closed pilon fracture have recently been reported. Xie et al. developed a prospective single-centre nomogram based on tourniquet use, preoperative hospital stay, BMI, albumin and high-sensitivity C-reactive protein, with a C-index of 0.838 in 417 patients [9]. Ke et al. subsequently proposed a retrospective model incorporating age, preoperative blood glucose, operative time, Tscherne classification and fracture classification, yielding AUCs of 0.898 and 0.880 in derivation and internal validation cohorts, respectively [4]. However, these nomograms relied exclusively on perioperative clinical variables and did not integrate composite systemic inflammatory indices or compare different inflammatory markers within the same cohort. In the present study, we systematically compared several preoperative systemic indices (SII, SIRI, NLR, PLR, HCLR and PAR), identified SIRI as the most informative biomarker through RCS and DeLong analyses, and incorporated SIRI into a parsimonious nomogram together with established clinical predictors. Despite being developed in a more contemporary population with a markedly lower SSI incidence (4.3%), our model maintained good discrimination (AUC 0.765 in the development cohort, 0.788 in temporal validation from the same center, and 0.779 in external validation from an independent center) and good calibration. Thus, although direct numerical comparisons of AUC across different cohorts should be interpreted with caution, our nomogram achieves discrimination that is comparable to previous pilon fracture models while explicitly accounting for host immune–inflammatory status, thereby enhancing the completeness and clinical interpretability of SSI risk prediction beyond mechanical and perioperative factors alone.

While direct clinical data confirming a link between SIRI and SSI are limited, several mechanistic and clinical studies in orthopaedic infection and bone pathology provide a biological rationale for this association. Experimental and translational work has shown that dysregulated innate immune activation within the bone microenvironment—characterized by sustained recruitment and activation of neutrophils and monocyte–macrophage lineages—can amplify tissue damage, impair angiogenesis, and create a niche that favours chronic infection or non-resolving inflammation [40–43]. Conversely, T-cell–mediated adaptive immunity plays a key role in orchestrating bone regeneration and controlling pathogen clearance; trauma- or surgery-induced lymphopenia has been linked to impaired immune surveillance and worse outcomes after orthopaedic injuries [44]. In this context, SIRI, which integrates circulating neutrophil, monocyte and lymphocyte counts, can be viewed as a composite marker of perioperative immune dysregulation—capturing the coexistence of exaggerated innate inflammation (neutrophilia/monocytosis) and relative adaptive immunosuppression (lymphopenia). This pattern is consistent with recent observations in patients with periprosthetic joint infection, where lower SIRI values and greater perioperative reductions were associated with improved infection control [39]. Taken together, these mechanistic and clinical data support the plausibility of our finding that elevated preoperative SIRI identifies patients with a systemic immune state that may be less capable of clearing bacterial contamination at the surgical site, thereby increasing the risk of SSI.

Beyond SIRI, our findings regarding patient-related factors and injury- or procedure-related characteristics emphasise that SSI after closed pilon fractures is driven by a multifactorial compromise of host resilience. These variables, previously reported in the literature, collectively indicate impaired systemic recovery capacity and increased injury severity. Elevated BMI may reflect a state of overnutrition or chronic low-grade inflammation, both of which have been linked to impaired immune responses and increased susceptibility to infection [45]. This association is particularly relevant in the context of acute trauma, where systemic inflammatory balance and immune competence are crucial for optimal recovery [46]. Therefore, nutritional and metabolic status, including BMI, should be carefully evaluated as part of preoperative risk assessment in such patients. A surgical delay of 6 days or more may reflect a need for preoperative stabilization in clinically complex or metabolically unstable patients—such as those with uncontrolled glycemia or malnutrition—during which systemic immune dysregulation and progressive local tissue deterioration may further elevate SSI susceptibility [15, 47]. A Tscherne grade 3 injury, despite the fracture being closed, denotes substantial soft tissue trauma that compromises local perfusion and structural integrity, creating an environment favorable to microbial colonization [4]. Prolonged operative duration may indicate surgical complexity or extensive injury, and also increases the duration of tissue exposure to potential contaminants [27, 48]. Elevated FBG, a known marker of poor glycemic control, impairs neutrophil function and collagen synthesis—both essential for infection control and wound healing [49]. It’s worth emphasizing that even the presence of multiple high-risk predictors does not guarantee the development of SSI, underscoring its multifactorial and non-deterministic nature.

The main strengths of this study include the large sample size and the use of RCS analysis to precisely evaluate the dose–response relationship between systemic inflammation biomarkers and the risk of SSI, which enabled the identification of the most informative biomarker and an optimal cutoff value prior to model construction. The combination of RCS-based biomarker selection with a clinically oriented nomogram design allowed us to move from statistical association to a tool with potential for implementation at the bedside.

Several limitations of this study should be acknowledged. First, the retrospective nature of the study inevitably limited the availability of certain variables, including surgeon-specific information (e.g., surgical expertise and intraoperative decision-making) and precise time-to-event data for SSI. These omissions may have introduced residual confounding and limited our ability to account for procedural heterogeneity and time-dependent risks. Second, patients were recruited from a tertiary orthopedic referral center, and a subset were transferred from remote hospitals. In such cases, surgical delays exceeding 6 days may have altered the baseline levels of systemic inflammation biomarkers due to evolving systemic responses to trauma [50]. However, we attempted to mitigate this potential bias by incorporating surgical delay as an independent variable in the predictive model. Third, although internal, temporal and external validation yielded favorable results, this study warrants further validation in large-scale, prospective, multicenter cohorts to confirm the generalizability of the nomogram. Fourth, for patients whose SSI was not diagnosed at our institution but was reported during telephone follow-up, only those who could provide medical documentation from other healthcare providers were included. While this strategy ensured diagnostic accuracy, it may have led to an underestimation of the true incidence of SSI. Fifth, our cohort did not include patients with open fractures or those requiring flap coverage for soft-tissue defects; therefore, the generalizability of our findings to the most severe soft-tissue injuries may be limited. Sixth, comparative external evidence involving SIRI and other systemic inflammatory indices specifically in fracture-related infections is currently lacking; therefore, our findings regarding the relative performance of SIRI versus other biomarkers are based on head-to-head analyses within this single cohort and should be interpreted with caution until confirmed in independent fracture populations. Last, standardized intraoperative contamination indicators (e.g., wound contamination grading or intraoperative cultures) were not available, which may lead to residual confounding.

## Conclusion

This study identified SIRI as a novel and independent systemic inflammation biomarker associated with SSI after closed pilon fracture. In addition, we developed and validated a predictive model that integrates SIRI with traditional clinical predictors (BMI, surgical delay, Tscherne classification, surgical duration, and FBG) to stratify SSI risk with reasonable accuracy in internal, temporal, and external cohorts. Together, identification of SIRI as a prognostic marker and development of a clinically oriented nomogram provide an initial framework for early SSI risk assessment and individualized perioperative decision-making in orthopedic trauma care. Nevertheless, these findings are hypothesis-generating and require confirmation in prospective multicenter studies before use in routine clinical screening.

## Supporting information

S1 Table Bootstrap validation of the prognostic value of SIRI ≥ 2.01 for SSI in the development cohort (B = 1,000 resamples). (DOCX)

S2 Table Pairwise DeLong tests comparing AUCs of systemic inflammatory indices for predicting SSI in the development cohort. (DOCX)

S1 Fig ROC curves of the full nomogram (with SIRI) and the baseline clinical model (without SIRI) for predicting SSI in the development cohort (AUC 0.765 vs 0.751). (TIF)

S2 Fig ROC curves of systemic inflammatory indices (SIRI, HCLR, NLR, SII, PLR and PAR) for predicting surgical site infection in the development cohort. SIRI and HCLR showed the highest AUCs (0.586), followed by NLR (0.564), SII (0.537), PLR (0.508) and PAR (0.485).
(TIF)

## References

[1] Mair O, Pflüger P, Hoffeld K, Braun KF, Kirchhoff C, Biberthaler P. Management of Pilon Fractures-Current Concepts. Front Surg. 2021;8:764232. doi: 10.3389/fsurg.2021.764232 35004835 PMC8732374

[2] Luo TD, Eady JM, Aneja A, Miller AN. Classifications in Brief: Rüedi-Allgöwer Classification of Tibial Plafond Fractures. Clin Orthop Relat Res. 2017;475(7):1923–8. doi: 10.1007/s11999-016-5219-z 28054323 PMC5449320

[3] Murawski CD, Mittwede PN, Wawrose RA, Belayneh R, Tarkin IS. Management of High-Energy Tibial Pilon Fractures. J Bone Joint Surg Am. 2023;105(14):1123–37. doi: 10.2106/JBJS.21.01377 37235679

[4] Ke C, Dong X, Xiang G, Zhu J. Risk factors and nomogram predictive model of surgical site infection in closed pilon fractures. J Orthop Surg Res. 2023;18(1):582. doi: 10.1186/s13018-023-04058-z 37553679 PMC10408134

[5] Esposito JG, van der Vliet QMJ, Heng M, Potter J, Cronin PK, Harris MB, et al. Does Surgical Approach Influence the Risk of Postoperative Infection After Surgical Treatment of Tibial Pilon Fractures?. J Orthop Trauma. 2020;34(3):126–30. doi: 10.1097/BOT.0000000000001655 32084089

[6] Potter JM, van der Vliet QMJ, Esposito JG, McTague MF, Weaver M, Heng M. Is the proximity of external fixator pins to eventual definitive fixation implants related to the risk of deep infection in the staged management of tibial pilon fractures? Injury. 2019;50(11):2103–7. doi: 10.1016/j.injury.2019.09.016 31530380

[7] Joveniaux P, Ohl X, Harisboure A, Berrichi A, Labatut L, Simon P, et al. Distal tibia fractures: management and complications of 101 cases. Int Orthop. 2010;34(4):583–8. doi: 10.1007/s00264-009-0832-z 19554328 PMC2903136

[8] Perencevich EN, Sands KE, Cosgrove SE, Guadagnoli E, Meara E, Platt R. Health and economic impact of surgical site infections diagnosed after hospital discharge. Emerg Infect Dis. 2003;9(2):196–203. doi: 10.3201/eid0902.020232 12603990 PMC2901944

[9] Xie L, Liu G, Wang X, Luo Z, Li Y, Wang X, et al. Development of a nomogram to predict surgical site infection after open reduction and internal fixation for closed pilon fracture: a prospective single-center study. J Orthop Surg Res. 2023;18(1):110. doi: 10.1186/s13018-023-03598-8 36793098 PMC9933287

[10] Cohen N, Kyin C, Norman D, Peskin B, Ghrayeb N, Perets I, et al. Risk factors for postoperative infection in patients after pilon fracture fixation. The Journal of Foot and Ankle Surgery. 2025;64(4):397–401. doi: 10.1053/j.jfas.2025.01.009 39862974

[11] Hu H, Zhang J, Xie X-G, Dai Y-K, Huang X. Identification of risk factors for surgical site infection after type II and type III tibial pilon fracture surgery. World J Clin Cases. 2022;10(19):6399–405. doi: 10.12998/wjcc.v10.i19.6399 35979296 PMC9294882

[12] Guo H, Song B, Zhou R, Yu J, Chen P, Yang B, et al. Risk Factors and Dynamic Nomogram Development for Surgical Site Infection Following Open Wedge High Tibial Osteotomy for Varus Knee Osteoarthritis: A Retrospective Cohort Study. Clin Interv Aging. 2023;18:2141–53. doi: 10.2147/CIA.S436816 38143487 PMC10748744

[13] Rutenberg TF, Gabarin R, Kilimnik V, Daglan E, Iflah M, Zach S, et al. Nutritional and Inflammatory Indices and the Risk of Surgical Site Infection After Fragility Hip Fractures: Can Routine Blood Test Point to Patients at Risk? Surg Infect (Larchmt). 2023;24(7):645–50. doi: 10.1089/sur.2023.118 37643292

[14] Zhao G, Chen J, Wang J, Wang S, Xia J, Wei Y, et al. Predictive values of the postoperative neutrophil-to-lymphocyte ratio, platelet-to-lymphocyte ratio, and lymphocyte-to-monocyte ratio for the diagnosis of early periprosthetic joint infections: a preliminary study. J Orthop Surg Res. 2020;15(1):571. doi: 10.1186/s13018-020-02107-5 33256763 PMC7708199

[15] Guo Y, Li C, Guo H, Wang P, Zhang X. Combining systemic inflammation biomarkers with traditional prognostic factors to predict surgical site infections in elderly hip fracture patients: a risk factor analysis and dynamic nomogram development. J Orthop Surg Res. 2025;20(1):43. doi: 10.1186/s13018-024-05446-9 39800738 PMC11727307

[16] Lord JM, Midwinter MJ, Chen Y-F, Belli A, Brohi K, Kovacs EJ, et al. The systemic immune response to trauma: an overview of pathophysiology and treatment. Lancet. 2014;384(9952):1455–65. doi: 10.1016/S0140-6736(14)60687-5 25390327 PMC4729362

[17] Xu T, Song S, Zhu K, Yang Y, Wu C, Wang N, et al. Systemic inflammatory response index improves prognostic predictive value in intensive care unit patients with sepsis. Sci Rep. 2025;15(1):1908. doi: 10.1038/s41598-024-81860-7 39809872 PMC11732978

[18] World Medical Association. World Medical Association Declaration of Helsinki: ethical principles for medical research involving human subjects. JAMA. 2013;310(20):2191–4. doi: 10.1001/jama.2013.281053 24141714

[19] Mathew G, Agha R, Albrecht J, Goel P, Mukherjee I, Pai P, et al. STROCSS 2021: Strengthening the reporting of cohort, cross-sectional and case-control studies in surgery. Int J Surg. 2021;96:106165. doi: 10.1016/j.ijsu.2021.106165 34774726

[20] Li C, Yang Z, Yang P, Li Z, Wang T, Xing B, et al. Association between post-trauma platelet-lymphocyte ratio and nonunion in patients with extremity fractures: a multicenter retrospective cohort study. Int J Surg. 2025;111(12):8943–52. doi: 10.1097/JS9.0000000000003265 40844879 PMC12695322

[21] Allegranzi B, Bischoff P, de Jonge S, Kubilay NZ, Zayed B, Gomes SM, et al. New WHO recommendations on preoperative measures for surgical site infection prevention: an evidence-based global perspective. Lancet Infect Dis. 2016;16(12):e276–87. doi: 10.1016/S1473-3099(16)30398-X 27816413

[22] Berríos-Torres SI, Umscheid CA, Bratzler DW, Leas B, Stone EC, Kelz RR, et al. Centers for Disease Control and Prevention Guideline for the Prevention of Surgical Site Infection, 2017. JAMA Surg. 2017;152(8):784–91. doi: 10.1001/jamasurg.2017.0904 28467526

[23] Moja L, Piatti A, Pecoraro V, Ricci C, Virgili G, Salanti G, et al. Timing matters in hip fracture surgery: patients operated within 48 hours have better outcomes. A meta-analysis and meta-regression of over 190,000 patients. PLoS One. 2012;7(10):e46175. doi: 10.1371/journal.pone.0046175 23056256 PMC3463569

[24] Zhao F, Chang VT, Cleeland C, Cleary JF, Mitchell EP, Wagner LI, et al. Determinants of pain severity changes in ambulatory patients with cancer: an analysis from Eastern Cooperative Oncology Group trial E2Z02. J Clin Oncol. 2014;32(4):312–9. doi: 10.1200/JCO.2013.50.6071 24366929 PMC3897256

[25] Riley RD, Ensor J, Snell KIE, Harrell FE Jr, Martin GP, Reitsma JB, et al. Calculating the sample size required for developing a clinical prediction model. BMJ. 2020;368:m441. doi: 10.1136/bmj.m441 32188600

[26] Zhang J, Lu V, Zhou AK, Stevenson A, Thahir A, Krkovic M. Predictors for infection severity for open tibial fractures: major trauma centre perspective. Arch Orthop Trauma Surg. 2023;143(11):6579–87. doi: 10.1007/s00402-023-04956-1 37418004 PMC10541339

[27] Ren T, Ding L, Xue F, He Z, Xiao H. Risk factors for surgical site infection of pilon fractures. Clinics (Sao Paulo). 2015;70(6):419–22. doi: 10.6061/clinics/2015(06)06 26106960 PMC4462573

[28] Croxford R. Restricted cubic spline regression: A brief introduction. 2016.

[29] Yu Y, Wen Y, Xia J, Dong G, Niu Y. Blood Cell Ratio Combinations for Diagnosing Periprosthetic Joint Infections: A Preliminary Study. Infect Drug Resist. 2025;18:635–45. doi: 10.2147/IDR.S489201 39911568 PMC11796439

[30] Pang Z, Liang J, Chen J, Ou Y, Wu Q, Huang S, et al. Systemic immune-inflammatory biomarkers combined with the CRP-albumin-lymphocyte index predict surgical site infection following posterior lumbar spinal fusion: a retrospective study using machine learning. Front Med (Lausanne). 2025;12:1590248. doi: 10.3389/fmed.2025.1590248 40809418 PMC12343696

[31] Wu W, Guo Z, Zhu P, Lv B, Mao Y, She C, et al. A novel indicator for predicting functional recovery in elderly hip fracture patients. Front Med (Lausanne). 2025;12:1538038. doi: 10.3389/fmed.2025.1538038 40182857 PMC11966028

[32] Fang Z, Gao B, Wang Z, Chen X, Liu M. Association of systemic inflammation response index with mortality risk in older patients with hip fracture: a 10-year retrospective cohort study. Front Med (Lausanne). 2024;11:1401443. doi: 10.3389/fmed.2024.1401443 38841577 PMC11150681

[33] Ding K, Shang Z, Sun D, Yang W, Zhang Y, Wang L, et al. The admission inflammatory biomarkers profile of elderly hip fractures and its association with one-year walking independence and mortality: a prospective study. Int Orthop. 2025;49(1):19–28. doi: 10.1007/s00264-024-06353-8 39466411

[34] Chen X, Fan Y, Tu H, Chen J. A Novel Nomogram Developed Based on Preoperative Immune Inflammation-Related Indicators for the Prediction of Postoperative Delirium Risk in Elderly Hip Fracture Cases: A Single-Center Retrospective Cohort Study. J Inflamm Res. 2024;17:7155–69. doi: 10.2147/JIR.S485181 39398226 PMC11471118

[35] Ling H, Li W, Huang Z, Lao Y, Deng G, Lu R, et al. Construction of a nomogram model for deep vein thrombosis in patients with tibial plateau fracture based on the Systemic Inflammatory Response Index. BMC Musculoskelet Disord. 2024;25(1):240. doi: 10.1186/s12891-024-07328-x 38539173 PMC10967150

[36] Fu M, Liu Y, Hou Z, Wang Z. Interpretable prediction of acute ischemic stroke after hip fracture in patients 65 years and older based on machine learning and SHAP. Arch Gerontol Geriatr. 2025;129:105641. doi: 10.1016/j.archger.2024.105641 39571498

[37] Fu Q, Zhang C, Yang Y, Teng R, Liu F, Liu P, et al. Correlation study of multiple inflammatory indices and vertebral compression fracture: A cross-sectional study. J Clin Transl Endocrinol. 2024;37:100369. doi: 10.1016/j.jcte.2024.100369 39308769 PMC11414683

[38] Liu B, Zhang Q. Systemic Immune-Inflammation-Based Biomarker and Fragility Fractures in People Living With HIV: A 10-Year Follow-Up Cohort Study in China. J Med Virol. 2024;96(11):e70052. doi: 10.1002/jmv.70052 39530247

[39] Vitiello R, Smimmo A, Matteini E, Micheli G, Fantoni M, Ziranu A, et al. Systemic Inflammation Response Index (SIRI) and Monocyte-to-Lymphocyte Ratio (MLR) Are Predictors of Good Outcomes in Surgical Treatment of Periprosthetic Joint Infections of Lower Limbs: A Single-Center Retrospective Analysis. Healthcare (Basel). 2024;12(9):867. doi: 10.3390/healthcare12090867 38727424 PMC11083165

[40] Zheng J, Yao Z, Xue L, Wang D, Tan Z. The role of immune cells in modulating chronic inflammation and osteonecrosis. Front Immunol. 2022;13:1064245. doi: 10.3389/fimmu.2022.1064245 36582244 PMC9792770

[41] Austermann J, Roth J, Barczyk-Kahlert K. The Good and the Bad: Monocytes’ and Macrophages’ Diverse Functions in Inflammation. Cells. 2022;11(12):1979. doi: 10.3390/cells11121979 35741108 PMC9222172

[42] Newman H, Shih YV, Varghese S. Resolution of inflammation in bone regeneration: From understandings to therapeutic applications. Biomaterials. 2021;277:121114. doi: 10.1016/j.biomaterials.2021.121114 34488119 PMC8545578

[43] Italiani P, Mosca E, Della Camera G, Melillo D, Migliorini P, Milanesi L, et al. Profiling the Course of Resolving vs. Persistent Inflammation in Human Monocytes: The Role of IL-1 Family Molecules. Front Immunol. 2020;11:1426. doi: 10.3389/fimmu.2020.01426 32754155 PMC7365847

[44] Hu W, Deng J, Su Z, Wang H, Lin S. Advances on T cell immunity in bone remodeling and bone regeneration. Zhejiang Da Xue Xue Bao Yi Xue Ban. 2024;53(4):450–9. doi: 10.3724/zdxbyxb-2023-0619 39183057 PMC11375490

[45] Abelleyra Lastoria DA, Ogbolu C, Olatigbe O, Beni R, Iftikhar A, Hing CB. The effect of combined malnutrition and obesity on trauma and orthopaedic surgery outcomes. Bone Joint J. 2024;106-B(10):1044–9. doi: 10.1302/0301-620X.106B10.BJJ-2024-0140.R2 39348912

[46] Dessouki O, Mahomed NN, Gandhi R. Metabolic abnormality and the proinflammatory state following hip joint surgery. International Journal of Clinical Rheumatology. 2011;6(3):347–58. doi: 10.2217/ijr.11.16

[47] Cheng X, Liu Y, Wang W, Yan J, Lei X, Wu H, et al. Preoperative Risk Factor Analysis and Dynamic Online Nomogram Development for Early Infections Following Primary Hip Arthroplasty in Geriatric Patients with Hip Fracture. Clin Interv Aging. 2022;17:1873–83. doi: 10.2147/CIA.S392393 36575659 PMC9790145

[48] Gowd AK, Bohl DD, Hamid KS, Lee S, Holmes GB, Lin J. Longer Operative Time Is Independently Associated With Surgical Site Infection and Wound Dehiscence Following Open Reduction and Internal Fixation of the Ankle. Foot Ankle Spec. 2020;13(2):104–11. doi: 10.1177/1938640019835299 30913923

[49] Yeramosu T, Satpathy J, Perdue PW Jr, Toney CB, Torbert JT, Cinats DJ, et al. Risk Factors for Infection and Subsequent Adverse Clinical Results in the Setting of Operatively Treated Pilon Fractures. J Orthop Trauma. 2022;36(8):406–12. doi: 10.1097/BOT.0000000000002339 34999622 PMC9253198

[50] Lord JM, Midwinter MJ, Chen Y-F, Belli A, Brohi K, Kovacs EJ, et al. The systemic immune response to trauma: an overview of pathophysiology and treatment. Lancet. 2014;384(9952):1455–65. doi: 10.1016/S0140-6736(14)60687-5 25390327 PMC4729362

