# Supplementary material for: Integrating systemic inflammation biomarker and clinical predictors for surgical site infection risk assessment in closed pilon fractures: A risk prediction model
Source: PLoS One. 2026 Apr 6;21(4):e0346298. doi: 10.1371/journal.pone.0346298 (PMC13052880; doi:10.1371/journal.pone.0346298)
Supplement: S1 Table — (DOCX) [file pone.0346298.s001.docx]

**Supplementary Table S1.** Bootstrap validation of the prognostic value of SIRI ≥ 2.01 for SSI in the development cohort (B = 1,000 resamples)

| Measure | Median | 2.5th percentile | 97.5th percentile |
| --- | --- | --- | --- |
| Odds ratio for SSI, SIRI ≥ 2.01 vs < 2.01 | 2.21 | 1.31 | 4.28 |
| AUC of dichotomised SIRI (≥ 2.01 vs < 2.01) | 0.595 | 0.534 | 0.656 |

Abbreviations: SIRI, systemic inflammation response index; SSI, surgical site infection; OR, odds ratio; AUC, area under the receiver operating characteristic curve.

Notes: For each of 1,000 bootstrap resamples of the development cohort, SIRI was dichotomised at the pre-specified cut-off of 2.01. The odds ratio for SIRI ≥ 2.01 versus < 2.01 was estimated using univariable logistic regression, and the corresponding AUC was calculated from the ROC curve. Values shown are the medians and 2.5th–97.5th percentile ranges of the bootstrap distributions (percentile method).
