# Supplementary material for: Integrating systemic inflammation biomarker and clinical predictors for surgical site infection risk assessment in closed pilon fractures: A risk prediction model
Source: PLoS One. 2026 Apr 6;21(4):e0346298. doi: 10.1371/journal.pone.0346298 (PMC13052880; doi:10.1371/journal.pone.0346298)
Supplement: S2 Table — (DOCX) [file pone.0346298.s002.docx]

**Supplementary Table S2.** Pairwise DeLong tests comparing AUCs of systemic inflammatory indices for predicting SSI in the development cohort

| Systemic Inflammation Biomarker | AUC | Z statistic vs SIRI | p-value* |
| --- | --- | --- | --- |
| **SIRI (reference)** | 0.586 | – | – |
| SII | 0.537 | 2.59 | 0.005 |
| NLR | 0.564 | 1.58 | 0.057 |
| PLR | 0.508 | 2.38 | 0.009 |
| HCLR | 0.586 | 0.01 | 0.496 |
| PAR | 0.485 | 1.83 | 0.033 |

Abbreviations: SIRI, systemic inflammation response index; SII, systemic immune-inflammation index; NLR, neutrophil-to-lymphocyte ratio; PLR, platelet-to-lymphocyte ratio; HCLR, high-sensitivity C-reactive protein-to-lymphocyte ratio; PAR, platelet-to-albumin ratio; AUC, area under the receiver operating characteristic curve; SSI, surgical site infection.

*p-values are from pairwise DeLong tests using SIRI as the reference ROC curve (null hypothesis H₀: AUC_SIRI ≤ AUC_marker; alternative hypothesis H₁: AUC_SIRI > AUC_marker).
